# Mind Your Manners! A Dataset and a Continual Learning Approach for Assessing Social Appropriateness of Robot Actions

**DOI:** 10.3389/frobt.2022.669420

**Published:** 2022-03-09

**Authors:** Jonas Tjomsland, Sinan Kalkan, Hatice Gunes

**Affiliations:** ^1^ Department of Computer Science and Technology, University of Cambridge, Cambridge, United Kingdom; ^2^ Department of Computer Engineering, Middle East Technical University, Ankara, Turkey

**Keywords:** human-robot interaction, social appropriateness, domestic robots, lifelong learning, Bayesian neural network

## Abstract

To date, endowing robots with an ability to assess social appropriateness of their actions has not been possible. This has been mainly due to (i) the lack of relevant and labelled data and (ii) the lack of formulations of this as a lifelong learning problem. In this paper, we address these two issues. We first introduce the Socially Appropriate Domestic Robot Actions dataset (MANNERS-DB), which contains appropriateness labels of robot actions annotated by humans. Secondly, we train and evaluate a baseline Multi Layer Perceptron and a Bayesian Neural Network (BNN) that estimate social appropriateness of actions in MANNERS-DB. Finally, we formulate learning social appropriateness of actions as a continual learning problem using the uncertainty of Bayesian Neural Network parameters. The experimental results show that the social appropriateness of robot actions can be predicted with a satisfactory level of precision. To facilitate reproducibility and further progress in this area, MANNERS-DB, the trained models and the relevant code are made publicly available at https://github.com/jonastjoms/MANNERS-DB.

## 1 Introduction

Social robots are required to operate in highly challenging environments populated with complex objects, articulated tools, and complicated social settings involving humans, animals and other robots. To operate successfully in these environments, robots should be able to assess whether an action is socially appropriate in a given context. Learning to navigate in the jungle of social etiquette, norms, verbal and visual cues that make up such a social context, is not straightforward. Little work has been done on allowing robots to obtain this ability and even for humans, it takes years to learn to accurately read and recognise the signals involved when determining the *social appropriateness* of an action.

The social robotics community has studied related problems such as socially appropriate navigation (Gómez et al., 2013), recognition of human intent (Losey et al., 2018), engagement (Salam et al., 2017), facial expressions and personality (Gunes et al., 2019). However, determining whether generic robot actions are appropriate or not in a given social context is a relatively less explored area of research. We argue that this is mainly due to the lack of appropriately labeled data related to social appropriateness in robotics.

**FIGURE 1:**
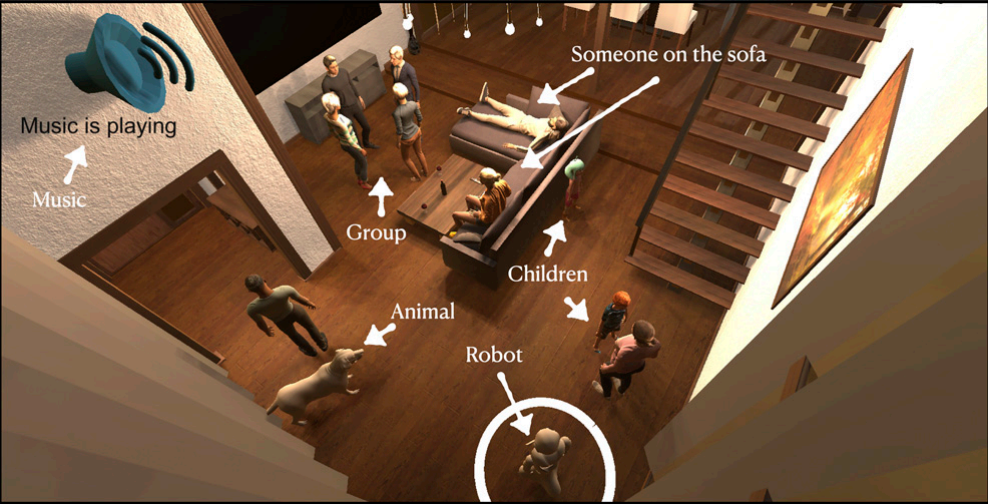
An example scene from the simulated living room environment. The robot (in circle) is expected to execute an action that is appropriate to the given social context.

To this end, we first introduce the Socially Appropriate Domestic Robot Actions Dataset (MANNERS-DB) that constitutes simulated robot actions in visual domestic scenes of different social configurations (see an example in Figure 1). In a similar vein to other robotics related dataset papers (Celiktutan et al., 2017; Lemaignan et al., 2018; Yang et al., 2021), we do not take a hypothesis formulation and testing approach. Instead, to be able to control but vary the configurations of the scenes and the social settings, MANNERS-DB has been created utilising a simulation environment by uniformly sampling relevant contextual attributes. The robot actions in each scene have been annotated by multiple humans along social appropriateness levels. Moreover, we train and evaluate a baseline Multi Layer Perceptron, as well as a Bayesian Neural Network that estimate social appropriateness of actions on MANNERS-DB, along with rich uncertainty measures enabled by the probabilistic approach. Finally, we formulate learning social appropriateness of actions as a *continual learning* problem, more precisely *task-incremental learning*, and propose a Bayesian continual learning model that can incrementally learn social appropriateness of new actions. Our experimental results show that the social appropriateness of robot actions can be predicted with a satisfactory level of precision. The aforementioned aspects of our work take robots one step closer to a human-like understanding of (social) appropriateness of actions, with respect to the social context they operate in.

## 2 Related Work

### 2.1 Social Appropriateness and HRI

Operating successfully in a social environment is already challenging for most people, let alone robots. The social cues and signals that need to be interpreted and acted upon are numerous and complex. However, some of the social rules and conventions that need to be followed and understood are similar for both humans and robots. A good starting point for this is the survey paper on social signal processing by Vinciarelli et al. (2009) that provides a compilation of the relevant cues associated with the most important social behaviours, including posture and usage of space.

In the context of group behaviour, Kendon (2009) proposed the *Facing-formation (F-formation) system of spatial organisation* where *F-formations* refer to the spatial patterns formed when people interact face-to-face. According to this framework, the space that an individual directs their attention to is called a transactional segment. When two or more people’s transactional segments overlap during an interaction, an F-formation with different configurations is formed (L-arrangement, face-to-face, side-by-side, semicircular, and rectangular arrangements). This framework has been widely adopted for automatic analysis of free-standing group interactions and we follow the same convention when analysing appropriate robot actions in the context of human groups.

When it comes to assessing how we use the space and the environment around us in social interactions, Hall et al. (1968) identified four concentric zones around a person, namely the intimate, the casual-personal, the socio-consultive and the public zone. He argued that the preferred interpersonal distance between the parties interacting is determined by the social relationship between them. The *intimate zone* is reserved for our closest relations which we embrace and physically touch. The *casual-personal zone*, is where we interact with friends and wider family. The *socio-consultive zone* is where acquaintances etc. are let in. And lastly, the *public zone* is where strangers and impersonal interactions often occur.

In the field of human-robot interaction, studies have shown that robots are treated differently than humans with respect to appropriate interpersonal distance and invasion of personal space. Evidence suggests that, when introduced to a robot, people prefer it to be positioned in what Hall et al. (1968) defines as the *social zone* and only after the first interactions they would feel comfortable allowing it into their *personal zone* (Hüttenrauch et al., 2006; Walters et al., 2009). Studies also show that these preferences change as people get used to the robot over time (Koay et al., 2007), and that these preferences are also dependent on robot appearance (Walters et al., 2008).

We note that majority of the existing works on robot behaviour toward and around people have focused on socially aware motion planning and navigation. Traditional approaches in this area rely on hand-crafted methods such as the work of Triebel et al. (2016) where the problem of socially aware navigation was broken down to detection, tracking and recognition of human relations and behaviour, followed by tailored motion planning. Similarly, human-aware navigation has been proposed by Ferrer et al. (2013) through the use of “social forces” interacting between humans and robot companions. On the other hand, modern machine learning approaches such as deep reinforcement learning (DRL) have also been utilised for socially compliant navigation. Using DRL, Chen et al. (2017) approached the challenge of socially appropriate motion planning by emphasizing what a social robot *should not do* instead of what it *should do*. Recently, new toolkits and guidelines for social navigation have been proposed (e.g., Baghel et al., 2020; Tsoi et al., 2020a,b).

Researchers have also examined how and when to engage humans appropriately in HRI situations (Walters et al., 2007). Michalowski et al. (2006) explored this based on sensory inputs indicating location, pose, and movement of humans in the environment. More recently, Gao et al. (2019) investigated how robots should approach groups of people in a socially appropriate way using deep learning. To the best of our knowledge, the perception and the machine-learning based recognition of social appropriateness of domestic robot actions has not yet been investigated.

Determining the social appropriateness of an action relies on determining the social context in which that action will be executed. Contextual understanding has been an important area of research in human-computer interaction (HCI) (Dey, 2001), and several works in the field of HRI have aimed to model context (Mastrogiovanni et al., 2009; Larochelle et al., 2011). Celikkanat et al. (2015) transferred the classical topic modelling method, Latent Dirichlet Allocation, and used it to model contexts. They placed probability distributions over objects and contexts instead of words and topics. All the above-mentioned works have mainly focused on context modelling and recognition, not what to do with that information when it becomes available. We take a different approach—instead of modelling context directly, we implicitly do so by determining appropriate robot actions given a context. Where the choice of context was based on that modelling the social appropriateness of actions in a living room of a home setting is more challenging and complex than for example a bedroom or a bathroom. We implement an end-to-end solution, mapping directly from the feature space obtainable through sensory inputs, to the social appropriateness of actions.

### 2.2 Continual Learning

Humans excel at continuously learning new skills and new knowledge with new experiences. This has inspired a new problem in machine learning, coined as lifelong learning (Thrun and Mitchell, 1995; Chen and Liu, 2016) or continual learning (CL) (Ring, 1994) and has for long been a difficult challenge for the deep learning community. Following the definitions of Lesort et al. (2020) we will use the term Continual Learning throughout this work which might overlap with other established terms such as Incremental Learning (Gepperth and Hammer, 2016) and Never-ending Learning (Carlson et al., 2010). In essence, continual learning covers the approaches that handle the challenges of learning new tasks or updating the old ones with a continuous stream of data, where the data distribution might change over time and where old data is not always available.

An important challenge in CL is to be able to retain the previously acquired knowledge while learning new ones. This is known as the catastrophic forgetting problem (McCloskey and Cohen, 1989; French, 1999). Unless appropriate measures are taken, learning from new experience tends to overwrite the previously learned associations. Over the years, many strategies have been devised against catastrophic forgetting (for reviews, see e.g., Thrun and Mitchell 1995; Parisi et al., 2019): Regularization-based, memory-based, and model-extension approaches. In regularization-based approaches, the destructive learning signals can be regularized by explicitly controlling which parameters (weights) are updated during learning (Sharif Razavian et al., 2014; Fernando et al., 2017) and how much they are updated (Kirkpatrick et al., 2017; Liu et al., 2021). Memory-based approaches, on the other hand, store previous experiences in memory and rehearse or indirectly use them in order to avoid forgetting them. To this end, the experiences themselves can be stored (Robins, 1993) or a generative model can be trained to generate pseudo-experiences (Robins, 1995) to rehearse experiences; or an episodic or semantic memory can be learned to retain information for longer terms and to interpret new experiences in the context of such a memory (Hassabis et al., 2017; Churamani and Gunes, 2020). Finally, in model-extension approaches, the model (the network architecture) can be extended itself to accommodate the required capacity for the new task or experience (Draelos et al., 2017). This can be achieved by adding new neurons (Parisi et al., 2017; Part and Lemon, 2017; Doğan et al., 2018a), layers (Rusu et al., 2016; Fernando et al., 2017) or both (Doğan et al., 2018b). Of course, hybrid approaches are also possible. For example, attention maps (Dhar et al., 2019) or embedded representations (Yu et al., 2020) for classes can be used to detect and mitigate catastrophic forgetting.

In this paper, we use a method that regularizes updates to parameters by looking at their uncertainties, following the approach of Ebrahimi et al. (2019). We extend this approach to predict epistemic and aleatoric uncertainties (with the method of Kendall and Gal 2017) and apply it to the continual learning of social appropriateness.

### 2.3 Continual Learning in Robotics

Continual learning is essential for robotics since robots interacting with the environment and the humans continuously discover new tasks, contexts and interactions. For a widespread use of robots, whenever needed, robots are expected to learn new tasks and skills, and to adapt to new experiences or contexts (Feng et al., 2019; Churamani et al., 2020; Kasaei et al., 2021; Ugur and Oztop, 2021).

There has been substantial work lately on addressing lifelong learning in robots, to enable lifelong learning in various robot capabilities, ranging from perception to navigation and manipulation (for reviews, see Churamani et al., 2020; Ugur and Oztop 2021; Feng et al., 2019; Kasaei et al., 2021). For example, Feng et al. (2019) benchmarked existing continual learning strategies for object recognition for a robot interacting continually with the environment. Liu et al. (2021) and Thrun and Mitchell (1995) studied lifelong learning for mobile robots navigating in different environments. Doğan et al. (2018a,b) introduced solutions for addressing lifelong learning of context in robots continually encountering new situations through their lifetimes. As a last example, Churamani and Gunes (2020) proposed a memory-based solution for continual learning of facial expressions that can potentially be used by a humanoid robot to sense and continually learn its user’s affective states.

Although these studies are promising, task-incremental learning within the social robotic aspect of HRI is less explored. What’s more, adapting to the behaviours of a robot in accordance with its users or new contexts is essentially a very practical setting of continual learning (Churamani et al., 2020). A detailed discussion on how this can be achieved in practice for various affective robotics and HRI problems, as well as the open challenges, is provided by Churamani et al. (2020).

### 2.4 Datasets Related to Social Appropriateness

There exist a couple of datasets for studying socially appropriate navigation in environments populated with objects and humans. For example, the Edinburgh Informatics Forum Pedestrian Database (Majecka, 2009) constitutes images of a large hall captured with a top-view camera. People walking by or across other people in the hall are captured by the camera. The navigation behaviors observed in the images can be used as targets for socially appropriate navigation behaviours in robots (Luber et al., 2012). The SocNav1 Social Navigation Dataset (Manso et al., 2020) contains different indoor settings with several humans and a robot navigating in the environment, which has been used for learning a map for socially appropriate navigation (Rodriguez-Criado et al., 2020).

Another dataset that is pertinent to our study is the CMU Graphics Lab Motion Capture Database (CMU, 2021) which contains 3D recordings of various types of human-human interactions (shaking hands, conversing, displaying non-verbal interactions etc.). This information has been utilized for learning personal comfort zones (Papadakis et al., 2013) which can be deployed on robots for navigation purposes.

Compared to the above mentioned datasets, MANNERS-DB is distinct as it considers a wider range of actions (cleaning, carrying objects etc.), modalities (includes sound) and social settings (includes children, pets, lying humans etc.). Therefore, our dataset makes it possible to study social appropriateness of robot actions in a more generic context than navigation.

### 2.5 Rich Uncertainty Estimates

Decision-making physical robots should provide insight into the uncertainty behind their actions, in particular when interacting with humans. In this work, we model two types of uncertainty, namely, aleatoric uncertainty describing the underlying ambiguity in the data and epistemic uncertainty reflecting the lack of or unfamiliarity with data. The two types were first combined in one model by Kendall and Gal (2017). They leveraged the practical dropout approach (Gal and Ghahramani, 2016) for variational Bayesian approximation to capture epistemic uncertainty, and extracted heteroscedastic aleatoric uncertainty by extending the model output to predict both a mean, $\hat{y}$ , and a variance, ${\hat{\sigma }}^{2}$ . Our work is different in that we implement the method in a continual learning application and use a BNN instead of the dropout approach. A social robotics implementation of this, making use of both aleatoric and epistemic uncertainty, is to our knowledge novel.

## 3 The MANNERS Dataset

Creating a real environment with simultaneously controlled and varied social configurations and attributes is difficult. Therefore, we developed a simulation environment to generate static scenes with various social configurations and attributes. The scenes were then labeled by independent observers *via* a crowdsourcing platform. As input data to our learning models we use a lower dimensional representation of the generated scenes using the variables presented in Table 1.

**TABLE 1:** The factors forming the 29-dimensional input to the learning models.

Feature	Variable type	Range
Operating within circle	Int	0 or 1
Radius of action circle	Float	0.5 → 3
Operating in the direction of an arrow	Int	0 or 1
Number of humans	Int	0 → 9
Number of children	Int	0 → 2
Distance to closet child	Float	0.4 → 6
Number of animals	Int	0 or 1
Distance to animal	Float	0.4 → 6
Number of people in a group	Int	2 → 5
Group radius	Float	0.50 → 1
Distance to group	Float	0 → 6
Robot within group?	Int	0 or 1
Robot facing group?	Int	0 or 1
Distance to 3 closest humans	3 x Float	0.3 → 5
Direction robot to 3 closest humans	3 x Float	0.0 → 360.0
Direction closest human to robot	Float	0.0 → 360.0
Robot facing 3 closest humans?	3 x Int	0 or 1
3 closest humans facing robot?	3 x Int	0 or 1
Number of people sofa	Int	0 → 2
Playing music?	Int	0 or 1
Total number of agents in scene	Int	1 → 11

### 3.1 Dataset Generation

#### 3.1.1 The Simulation Environment

The environment was developed in Unity3D simulation software (Unity, 2020). With Unity, we could generate a living room scene with various social configurations and attributes, involving groups of people, children and animals in different orientations and positions in space, music playing, robot facing the people etc. See an example scene with these aspects illustrated in Figure 1. The living room in which all the scenes are generated is part of a Unity Asset package from Craft Studios Apartment (2020). All avatars used to represent either people or animals are taken from Adobe’s Mixamo software Adobe (2020). Avatars are spawned into the living room scene as Unity Gameobjects, following a script written in the Unity compatible C# programming language.

#### 3.1.2 Scene Generation

Social appropriateness of robot actions can be investigated in numerous social contexts. In this work, we chose to focus on “visual” robot domestic actions that could potentially occur in a home setting as social robots are envisaged to be incorporated into our homes in the near future. Modelling the social appropriateness in the living room of such a home setting is more challenging and complex than for example a bedroom or a bathroom setting. Therefore, we chose the living room scenario as the context for MANNERS-DB and represented this context using and varying the features defined in Table 1. Thousand scenes were generated by uniformly sampling the factors listed in Table 1, which include the number of people, the number of groups with people, animals, their locations and orientations etc. Specific attention was paid to the uniform sampling of positions and orientations to ensure that the dataset contains a wide spectrum of proxemics (Hall et al., 1968; Kendon, 2009) configurations. In scenes where some features are not applicable, such as group-distance when no group exists in the scene, or interpersonal distance and directions when there is less than three people in a scene, the distance features were hard coded to a value of 50 and the direction features to a value of 1000.

#### 3.1.3 Robot Actions

We specifically consider the social appropriateness of the actions listed in Table 2. In total, 16 robot actions are investigated - all actions except for *Cleaning (Picking up stuff)* and *Starting a conversation* are investigated in two sets, based on whether they are executed in a region (within a circle surrounding the robot) or in a direction pointed by an arrow.

**TABLE 2:** The robot actions investigated in each scene.

Actions within a circle	Actions along an arrow
Vacuum cleaning	Vacuum cleaning
Mopping the floor	Mopping the floor
Carry warm food	Carry warm food
Carry cold food	Carry cold food
Carry drinks	Carry drinks
Carry small objects (plates, toys)	Carry small objects (plates, toys)
Carry big objects (tables, chairs)	Carry big objects (tables, chairs)
Cleaning (Picking up stuff)	Starting conversation

### 3.2 Annotation and Analyses

#### 3.2.1 Data Annotation

**FIGURE 2:**
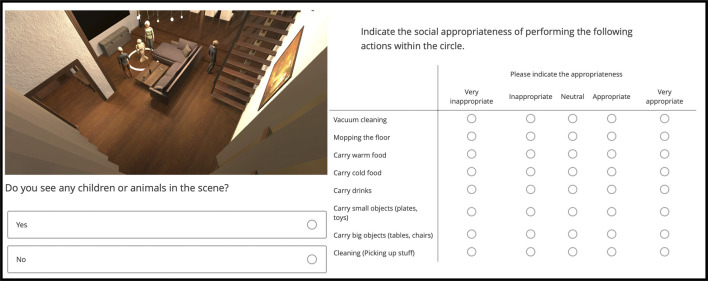
The annotation task as shown to the annotators on the crowd-sourcing platform. The page includes an image of the scene along with a honey-pot question (bottom-left) and questions around the appropriateness of robot actions.

The generated scenes were labelled for social appropriateness of the robot actions in the depicted domestic setting using a crowd-sourcing platform (Morris et al., 2014). The screenshot of what the annotators were presented with is depicted in Figure 2. Using this platform, we gathered 15 observer labels per scene, on a Likert scale from 1 to 5, where 1 represented “very inappropriate” and 5 “very appropriate.” The annotators constituted a varied group of English speakers. In order to avoid low-quality annotations, participants had to answer a honeypot question (similarly to Salam et al., 2017), that asked them whether there was an animal or child present in the scene (Figure 2). They were additionally requested to explain the reasons for the annotation they have provided *via* free-form sentences in a text box. Once the annotations have been obtained, we first analyze the quality of the annotations and what we can infer from them about the factors affecting social appropriateness of robot actions.

#### 3.2.2 Reliability

When collecting subjective opinions similarly to how it is done in our work, evaluating the inter-rater reliability is necessary to ensure that there is a sufficient level of consistency in the gathered labels (Hallgren, 2012). The inter-rater reliability measure also provides a good indication of agreement between annotators. To evaluate this, we compute two well-known metrics, intra-class correlation (ICC) (Shrout and Fleiss, 1979) for inter-agreement, and Cronbach’s *α* (Bland and Altman, 1997) for intra-agreement.

In Table 3, we present the ICC(1, 1) and ICC(1, *k*) values for all 16 actions, separated by scenes where the action is executed within a circle *versus* along an arrow. Note that all measures have a significance level of p < 0.001. Given the crowd-sourcing approach used in this work, we have *k* different raters (annotators) per scene (*k* = 15 in our case), all randomly sampled from a larger population of potential annotators. Knowing this, we examine ICC(1, 1) and ICC(1, *k*), per action over all scenes, backed by established guidelines for ICC (Koo and Li, 2016). ICC(1, 1), also called one-way random single score, gives an indication of the amount of agreement between any two raters. ICC(1, *k*), called one-way random averaged score, averages over the *k* raters’ independent scores.

**TABLE 3:** Inter-class correlation values for all actions over all scenes.

Actions	Intra-class correlation	
Actions within a circle	ICC(1,1)	ICC(1,k)
** **Vacuum cleaning	0.317	0.848
** **Mopping the floor	0.339	0.860
** **Carry warm food	0.068	0.465
** **Carry cold food	0.043	0.355
** **Carry drinks	0.048	0.378
** **Carry small objects (plates, toys)	0.087	0.533
** **Carry big objects (tables, chairs)	0.256	0.805
** **Cleaning (Picking up stuff)	0.192	0.740
Actions in the direction of an arrow	ICC(1,1)	ICC(1,k)
** **Vacuum cleaning	0.267 8	0.814
** **Mopping the floor	0.278	0.822
** **Carry warm food	0.048	0.378
** **Carry cold food	0.047	0.371
** **Carry drinks	0.042	0.346
** **Carry small objects (plates, toys)	0.078	0.503
** **Carry big objects (tables, chairs)	0.203	0.753
** **Starting a conversation	0.111	0.600

By looking at the intra-class correlations in Table 3, it is clear that single rater values [ICC(1, 1)] show lower correlations than when averaged over annotators [ICC(1, *k*)]. Going more into detail, we observe that the actions related to carrying food, drinks and small objects have substantially lower values than the rest of the actions. This could be explained by the nature of these actions being less intrusive and more ambiguous in terms of evaluating their appropriateness. The values obtained from ICC(1, 1) and ICC(1, *k*) were sufficient for the data to be used for machine learning modelling. If that had not been the case, previous work has shown that ranking-based methods can be used to remove low-quality contributors, i.e., going from 15 to 10 judgements but with higher agreement (Salam et al., 2016). This was not necessary in our case.

We also analyzed the reliability of the annotations using the Cronbach’s *α* metric (Bland and Altman, 1997), which tests the reliability of our crowd-sourced data by looking at internal consistency. For the actions-in-circle we obtain *α* = 0.885 and for actions-along-arrow, *α* = 0.851. According to Nunnally (1978), Cronbach’s *α* values over 0.70 are deemed as a good level of agreement.

#### 3.2.3 Perceived Social Appropriateness of Actions

**FIGURE 3:**
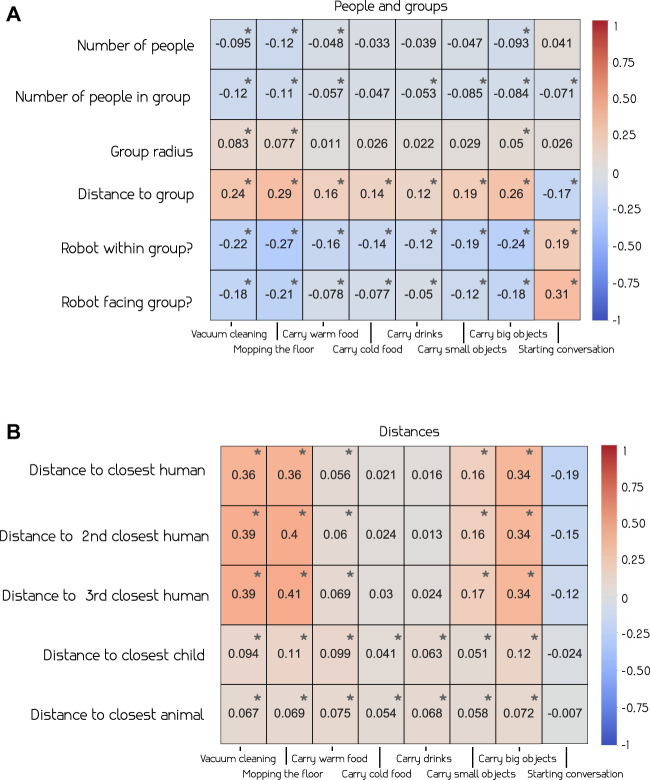
Pearson correlation (Freedman et al., 2007) between social appropriateness of actions and group related features **(A)** and distance related features **(B)**. We observe that certain group related features (e.g., distance to group) and distance-related features (e.g., distance to the closest human) have higher correlation to appropriateness – see the text for more details.

We explored the relation between the various factors and the social appropriateness of actions. Figure 3 provides the Pearson correlation coefficients for group-related (a) and distance-related features (b) respectively.

We observe that, in group related contexts, the number of people in the room, as well as in a group, seems to have slight negative correlation with the appropriateness of different actions. However, most of these are very close to zero, except for intrusive actions such as *vacuum cleaning* and *mopping the floor*. On the other hand, the distance from the robot to the group shows a more interesting relationship. We see that 7 out of the 8 robot actions positively correlate with distance, meaning that they might be deemed more appropriate when executed further away from the group. *Starting conversation* is the only action where this is not the case, which is reasonably determined as more appropriate when closer to a group. When looking at the impact of the robot being within the group, we observe opposite correlation values, indicating that all actions, but *starting conversation*, are less appropriate to execute when standing within a group.

The correlations of the distance related features (Figure 3B) indicate that most actions besides *starting conversation* are deemed more appropriate at a further distance. *Vacuum cleaning*, *mopping the floor* and *carrying big objects* have the highest correlation with distance features, further indicating that these might be viewed as intrusive and that the other actions are more appropriate to execute at a closer distance.

**FIGURE 4:**
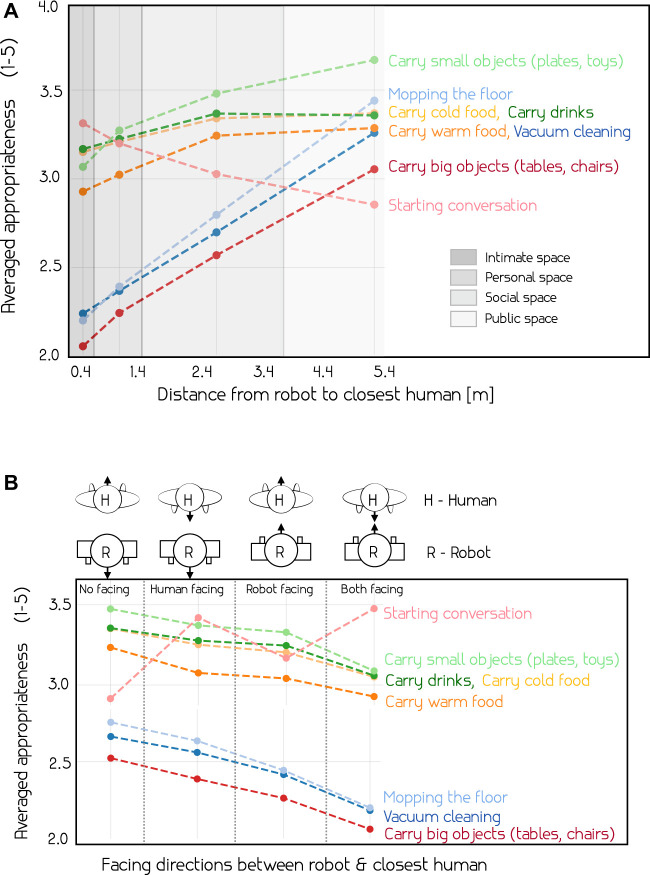
Average appropriateness of actions with respect to the distance to the closest person in the environment **(A**) and the orientation of the closest person in the environment **(B)**. We observe that distance to the closest human and the orientation between the human and the robot are significantly affecting appropriateness of certain actions – see the text for more details.

Building on the personal spaces of Hall et al. (1968), MANNERS-DB can be separated into parts based on where the robot executes an action with respect to the closest human. Figure 4A shows the labelled appropriateness of actions along the direction of an arrow with respect to the distance from the closest human, averaged over all samples. The different personal spaces in which the actions are executed in are indicated. We observe the same patterns here as in the correlation matrix from Figure 3, most actions become more appropriate further away from the closest humans, except *starting a conversation*. We also see the clear difference in reported appropriateness between intrusive actions such as *carrying big objects* and *vacuum cleaning*, and less intrusive alternatives like *carrying small objects* or *carrying drinks*. One surprising finding is that the appropriateness of *starting a conversation* is highest in the intimate space and decreases steadily with distance. Based on previous literature (Hüttenrauch et al., 2006), the expected result in this case would be that the reported appropriateness level starts quite low in the intimate space, peaks in the personal space and then slowly decreases with distance.

We investigate four different scenarios with respect to how the robot and the closest human face each other, see Figure 4B. The four different scenarios occur when: Neither the robot or the closest human face each other, when only one of them face the other (2 scenarios), and when both the robot and the closest human face each other. As expected, *starting a conversation* is deemed most appropriate when the closest human and the robot is facing each other. Interestingly, the relationship is flipped for the other actions, where the most appropriate situation is when neither the robot nor the human face the other. It is worth noting that the orientation has a subtle effect on the appropriateness of the less intrusive actions related to serving food and drinks, and a more substantial effect on *Vacuum cleaning*, *mopping the floor* and *carrying big objects*.

## 4 A Continual Learning Model for Social Appropriateness

In this section, we propose a continual learning model for learning social appropriateness of robot actions. For training our model, we use our MANNERS-DB dataset.

### 4.1 Architecture and Continual Learning Models

**FIGURE 5:**
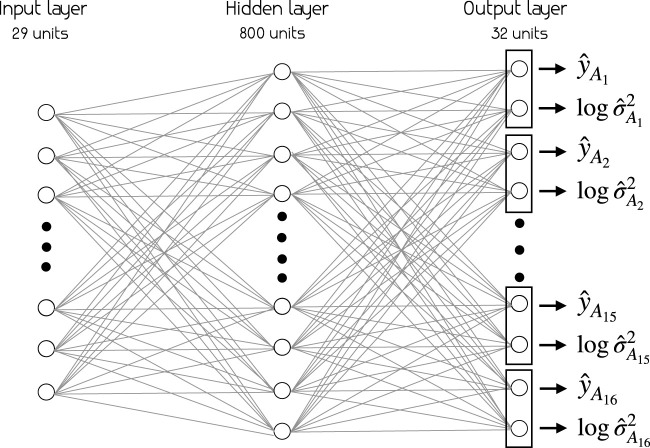
Neural network architecture for all models. The models take in the representation of the scene as a 29-dimensional vector (Table 1) and estimate social appropriateness of each action $\left({\hat{y}}_{A_{i}}\right)$ in range (1–5) as well as the uncertainty of estimation $\left(\log{\hat{\sigma }}_{A_{i}}\right)$ .

We experiment with two approaches, Multi Layer Perceptron (MLP) and Bayesian Neural Network (BNN), as baselines for estimating appropriateness ${\hat{y}}_{A_{i}}$ (in range 1–5) as well as the aleatoric uncertainty $\log{\hat{\sigma }}_{A_{i}}$ for each action *A*_*i*_. To evaluate and investigate the performance of our Bayesian continual learning framework, we conduct three experiments and compare the models’ ability to learn the social appropriateness of actions sequentially:

• Baseline (BNN and MLP):

Our baseline is the conventional MLP and BNN with the architecture shown in Figure 5, where no continual learning is used, but data for all actions are given at the same time to train the model.

• 2-tasks model (BNN-2CL and MLP2):

For the second experiment, we split the dataset into two. First training on the actions executed within a circle, followed by a new training session given samples with actions executed along the direction of an arrow. In other words, in this experiment, 2 tasks of 8 actions each are trained on sequentially.

• 16-tasks model (BNN-16CL and MLP16):

In the third experiment, the models are also given data sequentially, separated into parts for each of the 16 actions. Meaning, they are trained over 16 tasks of 1 action each.

All models share the same architecture, illustrated in Figure 5. The input vector is a 29-dimensional vector that consists of features detailed in Table 1. There is one hidden layer with 800 units and an output layer of 32 units (two for each action). These architectural choices are based on a thorough hyperparameter search investigating, among others, number of hidden layers and number of units in the hidden layer. The different models performed optimally with different architectures, but for the sake of a fair comparison we chose to use the same architecture for all of them.

Two of the models are implemented with active measures to handle catastrophic forgetting, the BNN-2CL and the BNN-16CL. They are extensions of the work of Ebrahimi et al. (2019), with some substantial modifications. In our work we deal with a regression task instead of classification, we use fully connected layers instead of convolution, and more importantly, we modify the output and loss function to obtain aleatoric uncertainty.

We would like to note that the continual learning problem in our paper is slightly different than in many other CL applications in that the distribution of the input data does not change significantly between two tasks, however, the labels do. For every task, the model is trained to predict the social appropriateness of a new set of actions. In more traditional applications, like sequentially learning to classify the handwritten digits of the MNIST dataset, both the input and the labels change. In other words, a handwritten five looks different from a four and they should be assigned to different classes. However, the approach taken in our work follows tightly with the overall human-like learning approach we have taken. As humans, we might face situations and contexts that we have seen before, but where we discover a new skill or develop our understanding related to that context, i.e. the input features are the same, but what we want to learn to predict changes.

### 4.2 Training the BNN

The inherent stochastic nature of BNNs leads to challenges at inference due to the intractability of the marginal probability, *p*(***Y*** | ***X***), which is needed to compute the posterior in Bayes theorem. To handle this, several approximate inference techniques have been proposed over the years (Hastings, 1970; Graves, 2011; Hernandez-Lobato et al., 2016), divided in two sub-groups: variational inference and sampling based methods. The Bayesian framework implemented in this work builds on the *Bayes-by-backprop* method introduced by Blundell et al. (2015), a back-propagation-compatible algorithm based on variational inference.

*Bayes-by-backprop* transforms the inference problem into an optimization problem by defining an approximate variational distribution, *q*_***θ***_(***ω***), and minimizing the Kullback-Leibler divergence (Kullback and Leibler, 1951) between this and the true Bayesian posterior on weights, *p*(***ω*** | ***X***, ***Y***). The resulting cost function is known as *variational free energy* or *expected lower bound* (ELBO), coined by Neal and Hinton among others (Saul et al., 1996; Neal and Hinton, 1998). To approximate this, Blundell et al. (2015) proposed a generalisation of the well-used Gaussian re-parameterisation trick (Opper and Archambeau, 2009; Kingma and Welling, 2013; Rezende et al., 2014), operating on stochastic weights instead of stochastic hidden units like previous work.

When combining the cost function given by *Bayes-by-backprop* with a regression loss, the following objective for the continual learning models are defined:

$$L\left(\theta \right)\approx \overset{M}{\underset{i=1}{\sum }}\left[\underset{\text{KL divergence}}{\underset{︸}{p_{1}\log q_{\theta }\left(\omega_{\left(i\right)}\right)\left.\right)-p_{2}\log p\left(\omega_{\left(i\right)}\right)\left.\right)}}+\underset{\text{Regression loss}}{\underset{︸}{\frac{p_{3}}{K}\overset{K}{\underset{j=1}{\sum }}\frac{1}{2}{\hat{\sigma }}_{j}^{-2}\|y_{j}-{\hat{y}}_{j}{\|}^{2}+\frac{1}{2}\log{\hat{\sigma }}_{j}^{2}}}\right].$$ (1)

Here, the first component controls variational approximation; the second component enforces the correctness of the predictions and estimates their uncertainty; *M* is the number of Monte Carlo samples; *K* represents the number of actions we predict the social appropriateness of during the training process – *K* is 16 for the BNN model, 8 for the BNN-2CL model and 1 for the BNN-16CL model; and *p*_1_ = 0.001, *p*_2_ = 0.001, *p*_3_ = 0.05 are empirically tuned constants.

### 4.3 Handling Catastrophic Forgetting

When undertaking continual learning, we need to deal with catastrophic forgetting. To prevent this, we use the uncertainty-guided continual learning strategy of Ebrahimi et al. (2019). This method proposes rescaling a “global” learning rate (*η*) to calculate a learning rate $\left(\eta_{\mu_{i}},\eta_{\sigma_{i}}\right)$ for each parameter (*ω*_*i*_ = (*μ*_*i*_, *σ*_*i*_)) according to the current variance *σ*_*i*_ of that parameter: $\eta_{\mu_{i}}\leftarrow \sigma_{i}\eta$ . Following Ebrahimi et al. (2019), we take $\eta_{\sigma_{i}}=\eta$ .

### 4.4 Estimating Uncertainties

We want to extract rich uncertainty estimates from the models. We do this through epistemic uncertainty (only BNNs) related to the lack of or unfamiliar data, as well as aleatoric uncertainty describing the underlying noise in the data. Examples of these two in our work could be high epistemic uncertainty for scenes with features that do not occur often in the training set, and high aleatoric uncertainty for scenes or actions where annotators had a high level of disagreement. Following the work of Kendall and Gal (2017) on uncertainty in computer vision, we extract these two types of uncertainty as follows:

$$\mathrm{Var}\left(y\right)\approx \underset{\text{Epistemic component}}{\underset{︸}{\frac{1}{T}\overset{T}{\underset{t=1}{\sum }}{\hat{y}}_{\left(t\right)}^{2}-{\left(\frac{1}{T}\overset{T}{\underset{t=1}{\sum }}{\hat{y}}_{\left(t\right)}\right)}^{2}}}+\underset{\text{Aleatoric component}}{\underset{︸}{\frac{1}{T}\overset{T}{\underset{t=1}{\sum }}{\hat{\sigma }}_{\left(t\right)}^{2}}},$$ (2)

where ${\hat{y}}_{\left(t\right)}$ and ${\hat{\sigma }}_{\left(t\right)}$ (for each action) are one out of *T* sampled outputs of stochastic forward passes through the BNN models. We take *T* = 100 following the literature (Kendall and Gal, 2017).

## 5 Experiments and Results

### 5.1 Implementation and Training Details

As mentioned, we kept the hyperparameters the same for all experiments to allow for a reasonable comparison in performance. Nevertheless, an extensive hyperparameter search was carried out to validate that this did not lead to a substantial drop in performance. For training, we used a batch size of 64, 200 epochs per task and an initial global learning rate *η* of 0.06. The learning rate *η* was decayed by a factor of 3 every time the validation loss stopped decreasing for 5 epochs in row, similar to traditional adaptive learning rate methods. Following the suggestions of Ebrahimi et al. (2019), in the BNN-2CL and BNN-16CL models we use 10 Monte Carlos samples to approximate the variational posterior, *q*_*θ*_(*ω*), and the initial mean of the posterior was sampled from a Gaussian centered at 0 with 0.1 in SD. The variable *ρ*, used to parameterise the SD of the weights, was initialised as -3. The two SD used in the scaled mixture Gaussian was set to 0 and 6, and the weighting factor for the prior, *π*, was set to 0.25.

Training on each task was done sequentially and the models’ weights were saved between tasks. This way, the change in performance, both the ability to predict accurate appropriateness and obtain sensible uncertainty measures, can be investigated with respect to the number of tasks the model has been trained on.

Training and Test Sets. For all three experiments, we split the dataset into training, validation and testing sets. The number of test samples, 100 scenes, are the same for all experiments, the training and validation sets are, however, separated differently to facilitate Continual Learning. The 650 scenes used for training and validation contain 9584 individual labelled samples. The validation part consist of 1000 samples for the baseline experiment, 400 samples per task (circle and arrow) for the BNN-2CL and MLP2 models and 100 per task (each action) for the BNN-16CL and MLP16 models. This means that the size of the training set for each experiment is approximately 8500 for the baseline, 4400 per task for the 2 task models and 500 per task for the 16 task models. It is worth noting that these differences in size of training set affect the comparative results obtained for each model as discussed in the next section.

### 5.2 Quantitative Results

The prediction results from the experiments are presented in Table 4. We see that all models generally estimate the appropriateness level (1–5) with low error (on average, with RMSE values lower than 1, for all models). Therefore we conclude that the social appropriateness of robot actions can be predicted with a satisfactory level of precision on the MANNERS-DB. When looking at the RMSE averaged over all actions, the values indicate that training on tasks sequentially impacts performance and that the active measures implemented in the BNN-2CL and BNN-16CL models work as intended. The BNN model had an average RMSE of 0.48, while the average RMSE for the BNN-2CL and BNN-16CL model were 0.53 and 0.63, respectively. The MLP on the other hand, performed better when training on all actions at once with an RMSE of 0.463, but had difficulties keeping up the performance when training sequentially over 2 and in particularly 16 tasks. It is important to note that for the 16 task experiment, the number of training samples per action is 1/8th of the number of training samples per action in the two other experiments. This would also affect the performance which makes it more difficult to pin what part of the increase in error is a result of continual learning and what part comes from the fewer number of training samples per action.

**TABLE 4:** Root-mean-squared error (RMSE) of predictions.

Actions	RMSE							
	MLP	MLP-2		MLP-16	BNN		BNN-2CL	BNN-16CL
Within a circle								
** **Vacuum cleaning	0.493	0.877		0.941	0.467		0.501	0.767
** **Mopping the floor	0.516	0.817		1.214	0.502		0.594	0.581
** **Carry warm food	0.472	0.796		0.617	0.445		0.448	0.810
** **Carry cold food	0.402	0.656		0.749	0.420		0.403	0.561
** **Carry drinks	0.390	0.771		0.790	0.402		0.485	0.733
** **Carry small objects	0.375	0.437		0.979	0.386		0.879	0.517
** **Carry big objects	0.533	0.734		1.271	0.497		0.520	0.665
** **Cleaning (Picking up stuff)	0.413	0.624		1.390	0.192		0.479	0.491
In direction of arrow								
** **Vacuum cleaning	0.547	0.573		1.014	0.555		0.591	0.750
** **Mopping the floor	0.541	0.551		1.063	0.542		0.602	0.664
** **Carry warm food	0.416	0.431		0.759	0.468		0.489	0.678
** **Carry cold food	0.441	0.446		0.883	0.477		0.495	0.526
** **Carry drinks	0.434	0.440		0.798	0.451		0.465	0.586
** **Carry small objects	0.417	0.425		0.431	0.431		0.464	0.548
** **Carry big objects	0.502	0.497		1.361	0.498		0.535	0.594
** **Starting a conversation	0.513	0.525		0.601	0.539		0.523	0.678
Mean over all actions	0.463	0.600		0.968	0.480		0.530	0.630

**FIGURE 6:**
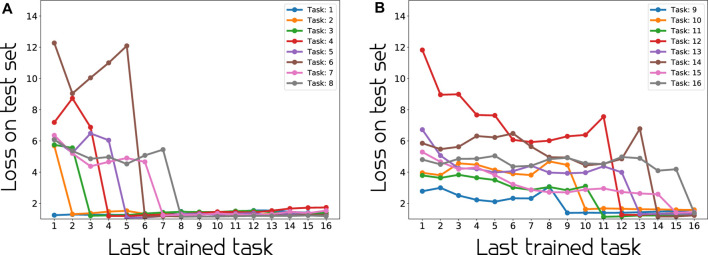
Per action performance on test data at different stages of continual learning. As expected, when training of a task starts, its loss decreases and the performances of previously trained tasks do not change significantly.

We provide an analysis of one of the continual learning model’s performance (BNN-16CL) in Figure 6. The figure shows that there is substantial difference in performance before and after training on a task/action. It also indicates clearly that before a task is trained on, its performance is affected by the training on other tasks. Looking at Figure 6A and task 6, we observe a good example of this, where the loss is increasing as the model is getting trained on other tasks, before dropping after being trained on the specific task at hand. Looking at Figure 6, we confirm that the loss on the test data for a specific task drops as the model gets trained on that specific task and thereafter, stays reasonably low and unaffected by the follow-up training process(es). This suggests that the model is able to handle catastrophic forgetting well.

### 5.3 Qualitative Results

The metrics presented above provide a good indication that all three BNN models perform well on unseen data. In this section we provide a qualitative evaluation of the predictions by taking a closer look at a number of representative scenes from the test set and the corresponding predicted social appropriateness of robot actions.

**FIGURE 7:**
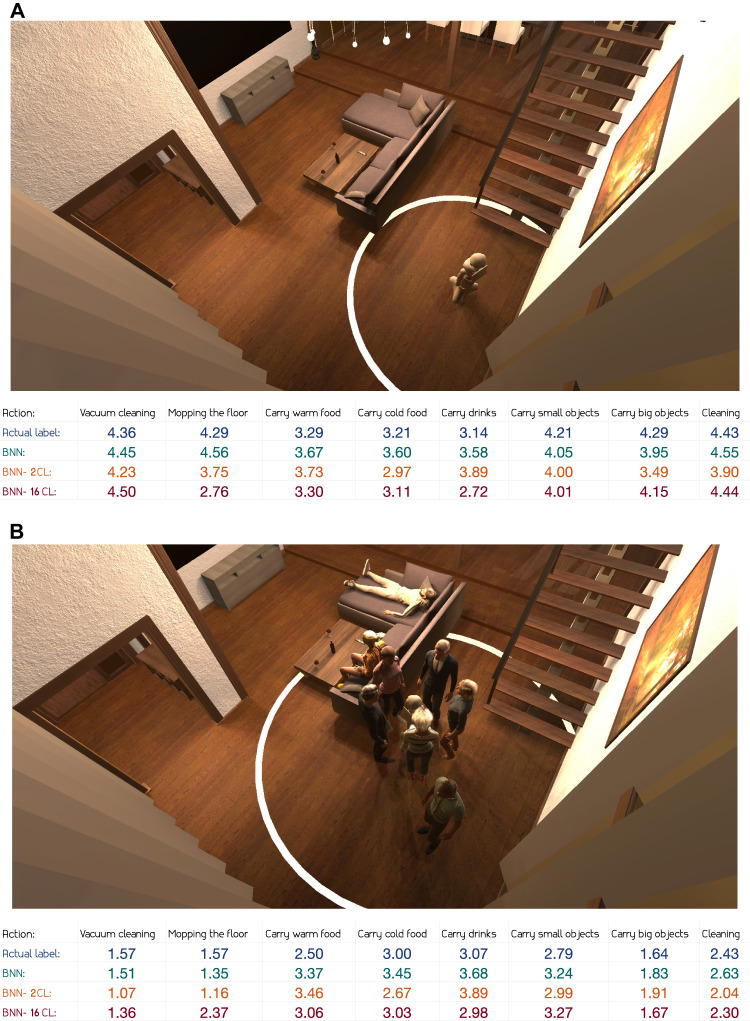
Predictions for test scene with no people **(A)** and with people, group and music **(B)**.

In Figure 7A, a scene where the robot is alone in the living room is presented. Here we observe that the labelled appropriateness level is high and the corresponding appropriateness prediction is also high for all actions. However, actions related to serving food seem to be deemed slightly less appropriate. This is expected given the lack of people in the room. Looking at explanations provided by annotators, this appears to be a valid reason. For instance, one of the annotator wrote: “There are no people around, so it seems more appropriate to be cleaning (and not to carry drink/food/etc). Noise is not a problem, since the room is empty,” and another annotator stated: “Can complete jobs but not provide services to others.”

Next, we take a closer look at a scene with more complex input features. In Figure 7B, we observe one man standing alone, a group of people standing around the robot and two people relaxing on the sofa. The robot’s working radius is quite large, encapsulating almost all the people in the room. Compared to the scene in Figure 7A, some of the actions, in particular the most intrusive ones, are both labelled and predicted with a lower level of appropriateness. This can be seen in the appropriateness levels of *vacuum cleaning*, *mopping the floor* or *carrying big objects*. We further observe from Figure 7 that both the annotators and the models deem less intrusive actions, such as *carrying cold food* and *carrying drinks*, as appropriate given the contexts. Interestingly, and perhaps to be expected given the small radius of the group surrounding the robot, *carrying warm food* is labelled as less appropriate than the two other serving-related actions. However, the models do not seem to be able to capture this subtle difference. Looking at the annotator explanations, the group radius seem to play an important role in this specific context: “Would be impolite to conduct certain tasks with so many people in such close proximity.” If we take a closer look at the predicted values for appropriateness from all models in Figure 7, and compare them to the true average of the annotators’ labels, the overall performance appears to be reasonable. The only values to stand out with a significant error are the BNN-16CL model’s predictions for *mopping the floor*.

**FIGURE 8:**
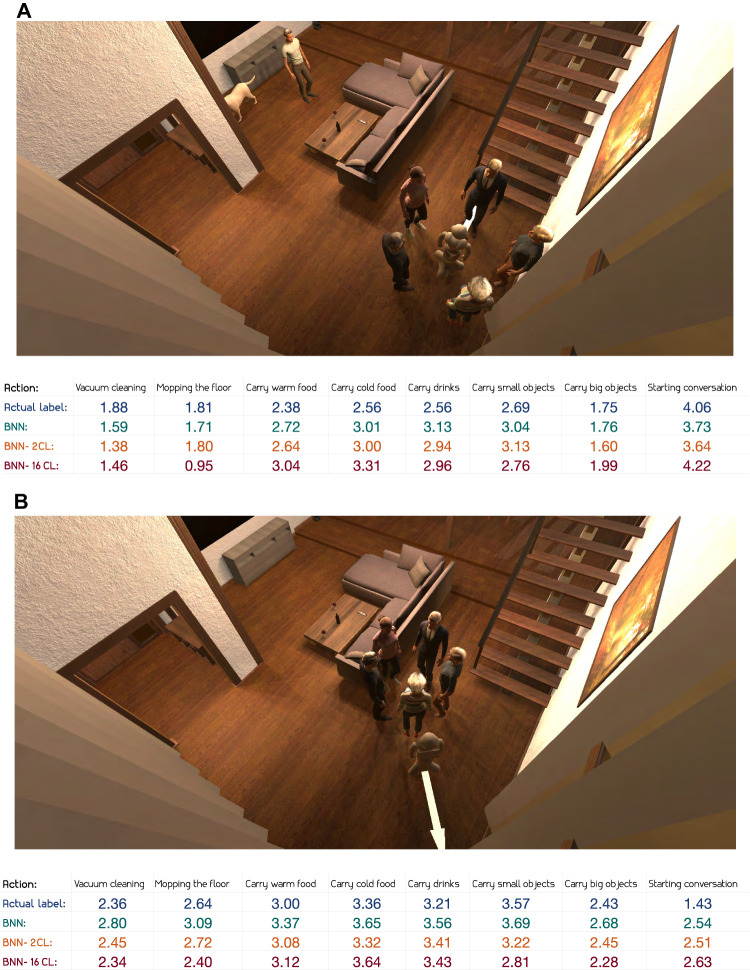
Predictions for test scene with actions along an arrow, with robot within group of people **(A**) and outside group of people **(B)**.

To conclude the qualitative evaluation of the predictions, two scenes where the robot executes actions along the direction of an arrow are presented. In Figure 8A, intrusive actions such as *vacuum cleaning* and *carrying big objects*, as well as *mopping the floor*, are labelled as inappropriate and the predicted values are also low for these actions. We observe that, similar to the scene in Figure 7B, actions related to serving food and drinks are predicted by the model as more appropriate than the intrusive actions. *Starting conversation* was not one of the actions investigated in the previously presented scene and as expected it is both labelled and predicted as appropriate given how the robot is facing one of the humans. Further insight can be obtained by looking at annotator explanations, where one reported: “Starting a conversation seems to be the number one action to do here. Therefore, the robot should not be doing any kind of cleaning nor carrying objects, especially big ones.” Another annotator responded that *starting conversation* would be the only appropriate option: “There is a person in the way. Only starting conversation is appropriate.” Overall, the models seems to capture the opinions of the annotators quite well.

In Figure 8B, the robot is positioned outside the group of people, facing away from them. As expected, the intrusive actions deemed inappropriate in the previous scene are now both labelled and predicted with slightly higher appropriateness levels. We observe that the model is more accurately predicting the appropriateness level of the actions related to carrying food or drinks. These values might seem questionably high given that the robot is moving away, however, annotator explanations from the scene in question indicate that carrying food away from the group is seen as appropriate: “He left the group. He could be bringing food or drinks left from them, but since the group is socializing, any other house chores should be avoided.” Also looking at Figure 8B, the labelled appropriateness level of *starting a conversation* is now considerably lower than when the robot was facing a human, and so are the predicted values. Annotators seem to believe that *starting a conversation* is less appropriate given this context. One annotator reported: “Moving away from a group of people, the robot definitely should not be trying to start a conversation.”

### 5.4 Analysis of Uncertainty Estimates

**FIGURE 9:**
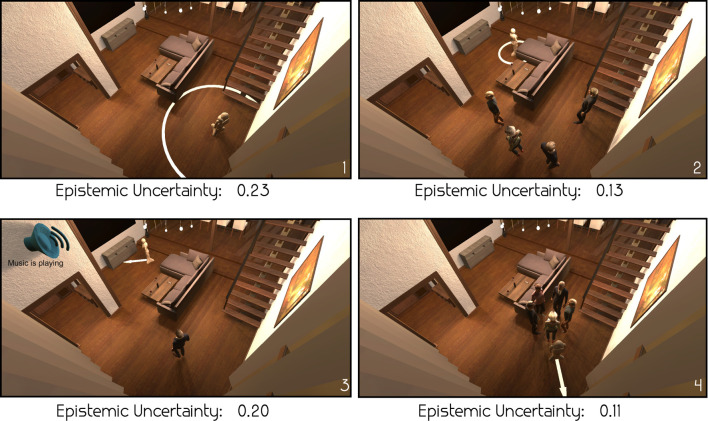
Four example scenes and their corresponding epistemic uncertainty estimate from the BNN-16CL model. As scenes 1 and 3 are less frequent in the dataset compared to scenes 2 and 4, we observe high epistemic uncertainty (indicating amount of familiarity) for scenes 1 and 3—see the text for more details.

We will now qualitatively evaluate the epistemic and aleatoric uncertainties for some example scenes in the test set. Since the MANNERS-DB dataset does not have significant differences in the input features for different actions, we compute the epistemic uncertainty per scene, averaged over all actions. However, we report the aleatoric uncertainty per action, as it should reflect the disagreement between annotators’ labels on each action. In Figure 9, we present four different scenes from the test set, along with their averaged epistemic uncertainty. Scenes 1 and 3 in Figure 9 show higher epistemic uncertainty in the prediction than scene 2 and 4. The reason for this might lay in the fact that the contexts simulated in scenes 1 and 3 are less frequent in the dataset than the ones showed in the two other scenes. Approximately 10% of the scenes in the dataset have no people in them, and the number of scenes with music playing is roughly the same. The contexts simulated in scenes 2 and 4 are much more common.

As we discussed in Section 2.5, one common approach for epistemic uncertainty estimation is the Monte Carlo (MC) Dropout method (Gal and Ghahramani, 2016). To see the reliability of epistemic uncertainties estimated by BNN and MC Dropout, we adapted the two epistemic uncertainty quality measures of Mukhoti and Gal (2018) for regression:

$$\begin{array}{ccc} p\left(accurate|certain\right) & = & \frac{\#accurate}{\#accurate+\#inaccurate\&certain}, \end{array}$$ (3)

$$\begin{array}{ccc} p\left(uncertain|inaccurate\right) & = & \frac{\#inaccurate\&uncertain}{\#inaccurate\&uncertain+\#inaccurate\&certain}, \end{array}$$ (4)

where we consider a prediction “accurate” if its RMSE is less than 0.1; and “certain” if the 0–1 normalized epistemic uncertainty (BNN or MC Dropout) is less than 0.5. For the two approaches, *p*(accurate|certain) is equal to 0.80 for BNN and 0.31 for MC Dropout; whereas *p*(uncertain|inaccurate) is equal to 0.89 for BNN and 0.05 for MC Dropout. We illustrate these with a number of examples in Figure 9 where MC Dropout estimates epistemic uncertainty as 0.111, 0.000, 0.019 and 0.012. Both the quantitative and qualitative results in Figure 9 suggest that BNN provides more reliable epistemic uncertainty estimates for the problem of predicting social appropriateness of robot actions.

**FIGURE 10:**
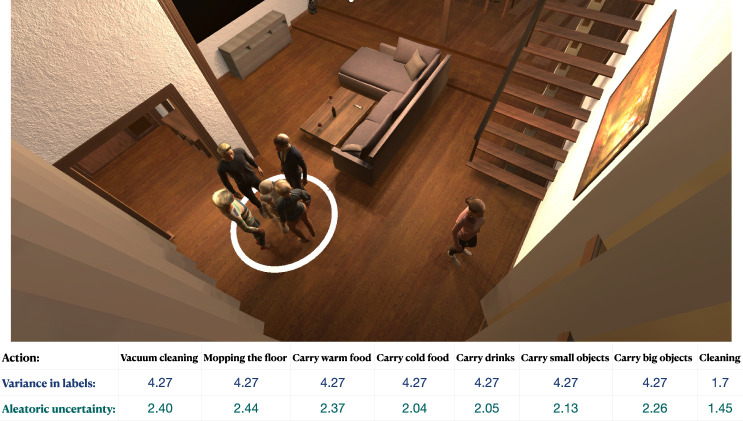
Example scene from test set with corresponding variance in annotator labels and aleatoric uncertainty per action. We observe that aleatoric uncertainty is high when the variance of labels is high, and vice versa. In other words, aleatoric uncertainty is able to capture the disagreement between the annotators in evaluations of social appropriateness of robot actions.

The aleatoric uncertainty should indicate annotator disagreement regarding the appropriateness of different robot actions. In our case, the dataset had high annotator agreement and therefore, annotator disagreement in aleatoric uncertainty was not observed. However, to further validate the models’ capability to capture aleatoric uncertainty, we increased the disagreement between annotators by artificially modifying the labels. In detail, we increased the variance in the labels by changing the annotators answers on the first 7 actions (for half of the dataset, they are set to one, and for the other half, to five) and leaving the original answer for the eighth action. By doing this we created a more distinct change in agreement levels between actions. See Figure 10 for an example scene with the corresponding variance in labels and predicted aleatoric uncertainty from the BNN-2CL model trained on the modified data. In this example scene, we can see that the aleatoric uncertainty follows the variance in the labels.

## 6 Conclusion and Future Work

In this work, we studied the problem of social appropriateness of domestic robot actions which, to the best of our knowledge, had not been investigated before. To this end, we first introduced a dataset with social appropriateness annotations of robot actions in static scenes generated using a simulation environment. The subjective appropriateness annotations were obtained from multiple people using a crowd-sourcing platform.

Our analysis of the annotations revealed that human annotators do perceive appropriateness of robot actions differently based on social context. We identified, for example, starting a conversation is perceived more appropriate if the robot is both close to the human and facing the human. We then formulated learning of social appropriateness of actions as a lifelong learning problem. We implemented three Bayesian Neural Networks, two of which employed continual learning. Our experiments demonstrated that all models provided a reasonable level of prediction performance and the continual learning models were able to cope well with catastrophic forgetting.

Despite its significant contributions, our work can be extended in various ways. For example, other environments, social settings and robot actions can be considered to study the social appropriateness of robot actions at large. This appears to be especially important for obtaining generalizable estimations from data-hungry learning models. In addition, our work’s simulation-based nature is likely to limit the ability of the models to catch nuances that are important when evaluating the appropriateness of actions in real-world scenarios. An interesting avenue of future research could include similar experiments with real-world social contexts. Moreover, our dataset contains textual annotations provided by users explaining the reasons behind their choices. This rich information can be leveraged for developing explainable models that can provide justifications for their social appropriateness predictions.

Going beyond the aforementioned future directions would entail generating dynamic scenes in which a robot is moving and/or generating scenes from robot’s first-person perspective, and obtaining relevant annotations for these scenes and movements. How to extend the research work and the results from the annotations obtained from the third-person perspective, as has been done in this paper, to the first-person perspective of the robot, would also be an interesting area to explore. Moreover, other CL methods (e.g., Ke et al., 2020) that handle catastrophic forgetting and the transfer of knowledge more explicitly among tasks.

## Data Availability

The original contributions presented in the study are included in the article/Supplementary Material, and the dataset, the trained models and the relevant code are made publicly available at https://github.com/jonastjoms/MANNERS-DB. Further inquiries can be directed to the corresponding author.
